# ECPC-ICP: A 6D Vehicle Pose Estimation Method by Fusing the Roadside Lidar Point Cloud and Road Feature

**DOI:** 10.3390/s21103489

**Published:** 2021-05-17

**Authors:** Bo Gu, Jianxun Liu, Huiyuan Xiong, Tongtong Li, Yuelong Pan

**Affiliations:** 1 School of Intelligent Systems Engineering, Sun Yat-sen University, Guangzhou 510006, China; gubo@mail.sysu.edu.cn (B.G.); liujx79@mail2.sysu.edu.cn (J.L.); 2 Guangdong Provincial Key Laboratory of Fire Science and Technology, Guangzhou 510006, China; 3 China Nuclear Power Engineering Co., Ltd., Shenzhen 518124, China; litongtong@cgnpc.com.cn (T.L.); panyuelong@cgnpc.com.cn (Y.P.)

**Keywords:** cooperative perception, intelligent vehicles, precise 6D pose estimation, sparse point cloud, roadside Lidars, point cloud registration, point cloud sparseness description

## Abstract

In the vehicle pose estimation task based on roadside Lidar in cooperative perception, the measurement distance, angle, and laser resolution directly affect the quality of the target point cloud. For incomplete and sparse point clouds, current methods are either less accurate in correspondences solved by local descriptors or not robust enough due to the reduction of effective boundary points. In response to the above weakness, this paper proposed a registration algorithm Environment Constraint Principal Component-Iterative Closest Point (ECPC-ICP), which integrated road information constraints. The road normal feature was extracted, and the principal component of the vehicle point cloud matrix under the road normal constraint was calculated as the initial pose result. Then, an accurate 6D pose was obtained through point-to-point ICP registration. According to the measurement characteristics of the roadside Lidars, this paper defined the point cloud sparseness description. The existing algorithms were tested on point cloud data with different sparseness. The simulated experimental results showed that the positioning MAE of ECPC-ICP was about 0.5% of the vehicle scale, the orientation MAE was about 0.26°, and the average registration success rate was 95.5%, which demonstrated an improvement in accuracy and robustness compared with current methods. In the real test environment, the positioning MAE was about 2.6% of the vehicle scale, and the average time cost was 53.19 ms, proving the accuracy and effectiveness of ECPC-ICP in practical applications.

## 1. Introduction

Vehicle 6DoF (Degrees of Freedom) pose estimation is an essential task in autonomous driving [1,2,3,4,5,6]. It is closely related to many critical self-driving subsystems, such as perception [7], decision [8,9], planning [10], control [1,11,12], and so on. Vehicle-mounted sensor positioning may have problems in scenes with sparse environmental features and dynamic changes in surrounding objects [13,14], such as campuses and ports.

**Figure 1:**
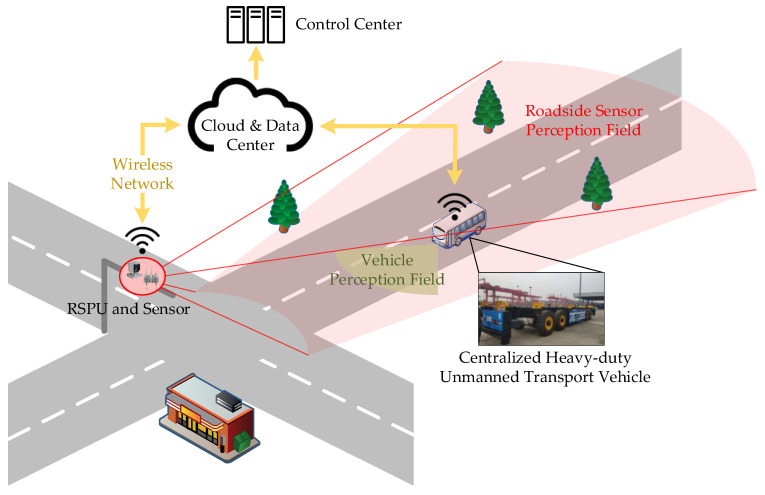
Vehicle pose estimation based on the roadside perception unit (RSPU) in a cooperative perception scene.

So with the development of intelligent transportation systems and cooperative perception technology [15,16], the research direction of unmanned vehicle pose estimation has gradually evolved to cooperative sensor positioning [3,17,18]. As shown in Figure 1, the precise vehicle positioning of roadside equipment can assist the automatic driving system to more accurately complete tasks such as planning and tracking [19], which plays a vital role in cooperative perception for intelligent vehicles. There are mainly two types of roadside sensor equipment: Lidar and camera [20]. Lidar is more widely used due to its advantages of accurate three-dimensional perception and resistance to environmental changes [21,22].

Challenges remain in vehicle pose estimation tasks using roadside Lidars. The roadside Lidar has an excellent deployment position, but the point cloud still has obscuration and points missing problems due to the fixed measurement view. More importantly, the resolution of a specific roadside Lidar is usually a fixed value. The vehicle point cloud is very sparse at long measurement distances (usually dozens of points). Sparse point cloud input information causes a challenge to the accurate 6D pose estimation task.

Many algorithms have been proposed for vehicle pose estimation based on the point cloud. The current algorithms can be roughly divided into two categories [23]: model-based approaches and model-free methods. The former has higher accuracy, and most of them need a dense point cloud to extract features for matching calculations. Researchers made breakthroughs in feature extraction [24,25,26,27], corresponding points matching [28,29,30,31,32,33], iterative calculations [34,35,36,37], and so on. On the contrary, model-free methods had fewer requirements for point cloud data, good generalization, but worse positioning accuracy. Researchers proposed different statistical calculation methods [38,39] and optimization functions for vehicle shape fitting [40,41,42,43,44,45,46]. Most of them operated fitting calculations in the two-dimensional space and performed better on vehicle point clouds with specific shapes (L shape or I shape [41]). The point cloud sparseness also caused the point cloud hard-to-describe vehicle shapes, resulting in a greater probability of solving a local minimum solution.

In this paper, a precise target vehicle 6D pose estimation algorithm was proposed for a sparse point cloud from roadside Lidars, named Environment Constraint Principal Component-Iterative Closest Point (ECPC-ICP). Aiming at the sparse point clouds, ECPC was a method for vehicle initial pose estimation that took advantage of road geometry information to achieve a stable pose solution. The ICP was then fused to achieve an accurate output. The ECPC-ICP combined model-based and model-free ideas to achieve a stable and accurate pose estimation.

Specifically, the proposed method first obtained the road normal information through ground fitting in the preprocessing stage. It calculated the maximum eigenvector of the normalized autocorrelation matrix of the clustered vehicle point cloud matrix. The vehicle’s local coordinate system was obtained as the initial pose result by fusing the above two spatial information through the vector outer product calculation. The target vehicle dense point cloud template was used to obtain accurate 6D pose through point-to-point ICP [47,48] registration.

In addition, according to the roadside Lidar measurement characteristics, the point cloud sparseness description was defined for quantitative verification in this paper. The proposed method was tested in a simulated environment and a real environment, proving that ECPC-ICP had better accuracy and robustness of 6D pose estimation than the current algorithms.

To summarize, the major contributions of this paper were two-fold:

- We proposed a novel method ECPC for initial pose estimation under sparse point clouds. ECPC integrated road normal information into global features of the sparse point cloud and achieved a robust solution to the initial pose.
- We proposed a point cloud sparseness description according to the measurement characteristics of roadside Lidar for quantitative experimental verification. The experiment was developed under point clouds with different sparseness, which proved the effectiveness of the proposed ECPC-ICP algorithm.

The rest of this paper was organized as follows: A related research overview was described in Section 2. The proposed ECPC-ICP pose estimation method was introduced in Section 3. Experimental design and results were described in Section 4. The conclusion was finally shown in Section 5.

## 2. Related Works

This section reviewed the model-based methods and model-free methods for intelligent vehicle 6D pose estimation.

### 2.1. Model-Based Methods

Most model-based methods rely on corresponding points to calculate the target pose. Corresponding points are usually selected by comparing local descriptors. The ECV feature [28] was proposed to establish the corresponding point relationship between the measured point cloud and the target point cloud. The boundary information was well extracted and processed in the ECV descriptor. Further, point cloud feature descriptors such as NARF [24], SHOT [25], PFH [26], FPFH [27] were also developed and used for model-based target pose detection, which realized feature matching and posed solving for dense point clouds.

A self-supervised learning model was proposed in [29] to learn local descriptors for registration and achieved better precision performance. Although the methods of establishing point correspondences by descriptors achieved higher accuracy, a common limitation was that they only worked normally for dense point clouds. Locally missing and large noise in the sparse point cloud would cause incorrect matching of the point pairs.

Researchers also made explorations in corresponding points matching. The coherent point drift method [31] and quick voting scheme on oriented point pair features [33] were also raised and improved the performance of convergence in the presence of noise, outliers, and missing points. The occlusion situations were handled well, but the performance was not stable for the sparse point cloud. Wang, R.D. [34] reformed the RANSAC algorithm with a novel framework multi-layer RANSAC, which improved the robustness of the point cloud registration in urban complex dynamic environments. However, it was difficult to achieve the same accuracy as the registration algorithms.

Based on FPFH, the SAC-IA [35] algorithm obtained the initial pose through statistical calculation of the spatial transformation relationship between randomly selected corresponding point pairs. ICP [47,48] or NDT [49] could also be added to improve the accuracy of pose estimation. Although the robustness of SAC-IA was improved by the two-stage registration, the initial pose solution based on the descriptor still required dense point cloud input. Mo, Y.D. [36] segmented the target point cloud by a regional growing algorithm. The point cloud boundary feature was extracted to calculate the target pose through ICP registration, which was the main improvement but also the main limitation. 

These explorations used point cloud descriptors to establish corresponding point pairs and then used the correspondences to perform a pose calculation. Although the model-based method could obtain higher accuracy, dense point cloud data was required to ensure that the descriptors were available. Under the condition of the sparse point cloud obtained by the roadside Lidars, the local descriptor information was insufficient, making it difficult for the above methods to converge stably.

### 2.2. Model-Free Methods

Model-free methods are usually based on the vehicle geometric feature fitting or point cloud statistical feature. Based on PCA [38], four PCA algorithms [39] for vehicle attitude regularization were proposed, and the superiority of Center-of-Gravity PCA and Continuous-PCA was verified through experiments. The fast calculation and superior stability were achieved, but large errors existed in the pose estimation tasks.

Since most point clouds in-vehicle pose estimation scenes contained the information of two-vehicle sides [41], in [40,42,43], the L-shape fitting algorithms based on vehicle morphology analysis were proposed for vehicle pose estimation. Zhang et al. [40] formulated the L-shape fitting as an optimization problem. Three optimization models for L-shape fitting were proposed. Among them, the Closeness Criterion had better accuracy and robustness than Area Criterion and Variance Criterion. The experiment proved that the Closeness Criterion obtained stable and accurate pose results for L-shaped vehicle point cloud data. However, the solution was the three-degree-of-freedom pose and was susceptible to interference from vehicle morphological noise.

Moreover, vertex and corner points were detected in [43] to get a more accurate pose estimation. The DATMO system was established and applied to autonomous driving tasks in [44]. It obtained the vehicle pose and achieved target tracking by calculating the line and corner features of the 2D target point cloud. Geometric features were well extracted to solve stable poses. So those methods were difficult to work properly in scenes with weaker features. In [41,45,46], the vehicle point cloud was fitted with a rectangular shape. The best-fitting rectangle was obtained by minimizing the distance between the point cloud and the rectangle boundary, and the line shape point cloud could also be well fitted. They were also susceptible to interference from vehicle morphological noise.

Although the model-free methods required low point cloud data density and had better robustness, their fitting results were relatively inaccurate. Additionally, it was difficult to maintain a stable solution when the vehicle point cloud shape changed. For example, the shape of the vehicle point cloud would be affected by the side mirror points [40]. More importantly, model-free methods mainly calculated the target vehicle pose in a 2D space, making the 6D pose unavailable.

## 3. Pose Estimation Considering Road Constraint

In this section, the proposed precise 6D pose estimation method ECPC-ICP with road information constraint was introduced. The 6D pose description described in Section 3.1 was the problem modeling of ECPC-ICP registration. The adopted point cloud preprocessing and segmentation method was presented in Section 3.2, where the road feature constraint was calculated. The proposed ECPC-ICP registration pipeline for sparse point clouds obtained by roadside Lidar was demonstrated in Section 3.3.

### 3.1. 6D Pose Estimation Modeling

In pose estimation tasks, the objective is usually described as finding the rotation transformation matrix $R\in {\mathbb{R}}^{3\times 3}$ and translation matrix $t\in {\mathbb{R}}^{3}$ that minimizes the squared distance between corresponding point clouds:

(1) $$\hat{R},\hat{t}=\arg\underset{R,t}{\min}{\left\|\left(R\cdot P_{s}+t\right)-P_{t}\right\|}^{2}$$

where $P_{s}$ represents the source point cloud, and $P_{t}$ represents the target point cloud.

In the global coordinate system, the 6D pose of the target vehicle can be expressed as a 6-dimensional vector: $\left[P_{x},P_{y},P_{z},R_{x},R_{y},R_{z}\right]$, where $P_{x},P_{y},P_{z}$ represent the coordinates of the vehicle center position. $R_{x},R_{y},R_{z}$ represent the orientation of the longitudinal vehicle axis in the form of a unit vector (the initial orientation value was [1,0,0]). The 6D pose could also be equivalently expressed by R and t. The corresponding relationship was:

(2) $${\left[P_{x},P_{y},P_{z}\right]}^{T}=t$$

(3) $${\left[R_{x},R_{y},R_{z}\right]}^{T}=R\cdot {\left[1,0,0\right]}^{T}$$

### 3.2. Point Cloud Preprocessing and Segmentation

In the cooperative vehicle 6D pose estimation task, the roadside Lidar had a stable viewing angle. Several methods were developed for background point filtering based on roadside Lidars. Wu et al. [50] fused 2500 frames of roadside data to generate background information. Then the point cloud was divided into spatial voxels, and the background points were filtered by density contrast. However, it was difficult to balance the accuracy and efficiency of filtering [51]. Background filtering methods based on laser channel information were proposed in [52,53], yet were only available for structured point clouds.

**Figure 2:**
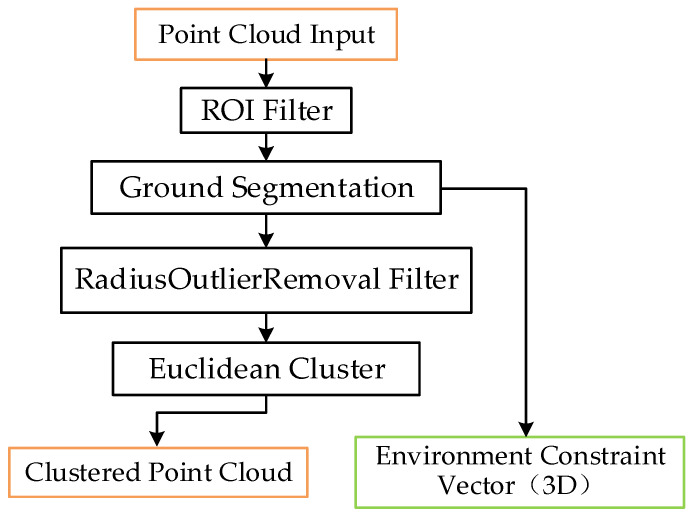
Adopted preprocess and segmentation method.

Considering the uniqueness of roadside point cloud data, this paper proposed a preprocess and segmentation procedure based on [54], which is shown in Figure 2. 

This paper preprocessed the point cloud through ROI (Region of Interest) filtering to remove long-distance background points. In this paper, the selected horizontal ROI was the sum of the vehicle driving area, i.e., the road area. Considering the vehicle size, the vertical ROI was set from −1 m to 6 m (ground level was considered as 0 m) to completely retain the ground points and vehicle points. The discriminants were designed for point cloud filtering based on ROI.

Then the RANSAC algorithm [55] was used for road fitting according to [56]. In this paper, RANSAC was run for one road plane with a 0.1 m threshold due to the detecting noise. The obtained road point cloud data could be expressed as a 4-dimensional vector G:

(4) $$G=\left[GN_{x},GN_{y},GN_{z},d\right]$$

which means the space plane represented by equation $GN_{x}\cdot X+GN_{y}\cdot Y+GN_{z}\cdot Z+d=0$, where X, Y, Z represents the coordinates of the point. Road normal features are retained as the environmental constraint information EC, which was one of the inputs in the following subsection. The specific form was:

(5) $$\mathrm{EC}={\left[GN_{x},GN_{y},GN_{z}\right]}^{T}$$

It is worth noting that the road scene involved in this paper was flat, and the road information could be well extracted by the RANSAC algorithm. The proposed ECPC-ICP method focused on the road normal features of the vehicle driving area and was not limited by the road fitting algorithm. Other road fitting algorithms, such as cloth simulation [57] and slope-based filtering [53] could also well support our ECPC-ICP pose estimation method.

In this paper, the RadiusOutlierRemoval filter [58] and the Euclidean cluster [59] were adopted for environmental noise removing and target point cloud clustering, respectively. In the RadiusOutlierRemoval filtering, points whose neighbors were less than the threshold were removed. This reduced the interference of isolated points after ground segmentation on the subsequent clustering. The search radius and neighbor threshold were set to 0.5 m and 3, respectively.

In the Euclidean cluster, the radius threshold $r_{th}$ was set to an appropriate value according to the actual situation and vehicle size. All the points within the range $r_{th}$ of each point in a cluster were classified into the same cluster. The process of iteration was repeated to complete the spatial division. Then the clustered vehicle point clouds were calculated.

**Figure 3:**
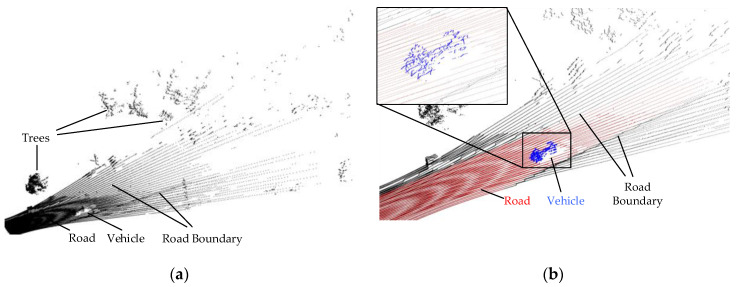
The typical result of the proposed preprocess and segmentation procedure. (**a**) The input point cloud obtained by the roadside Lidar; (**b**) The point cloud result after preprocessing and segmentation procedure, where the red points represent the ground points extracted by RANSAC, and the blue points are the vehicle points clustered by the Euclidean cluster, and the black points are the filtered background points.

Figure 3 shows the typical effect of the proposed preprocess and segmentation procedure. The blue points completely represent the vehicle shape and contain only a few background points, indicating an accurate clustering result.

### 3.3. ECPC-ICP Pose Estimation Method

**Figure 4:**
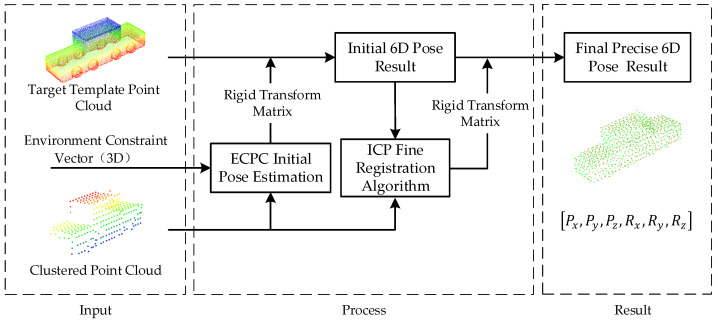
Proposed ECPC-ICP registration method.

This paper proposed a two-stage registration method ECPC-ICP, combining the ECPC initial pose estimation and ICP registration to solve the accurate target vehicle 6D pose. The ECPC-ICP pipeline is shown in Figure 4. The input to ECPC-ICP was three parts: completed vehicle point cloud template matrix $PC_{T}\;\left(3\times N_{T}\right)$, environment constraint vector EC (road normal features in Section 3.2), and clustered point cloud matrix $PC_{C}\;\left(3\times N_{C}\right)$ from Section 3.2.

The template preparing method was introduced in Section 3.3.1. The proposed ECPC initial pose estimation with road feature constraint was explained in Section 3.3.2. The final pose calculation based on point-to-point ICP registration was elaborated in Section 3.3.3.

#### 3.3.1. Target Template Preparing

As for the point cloud template $PC_{T}$, this paper used the vehicle’s overall point cloud template instead of the multi-view-point cloud template library, which avoided feature misidentification and reduced the difficulty of template generation. Simultaneously, compared to feature-based pose estimation methods, the use of overall point cloud registration reduced the feature analysis errors and improved the accuracy of pose solutions. $PC_{T}$ could be obtained by sampling the visible part of the CAD model or splicing from the scanned target multi-view-point clouds, which was elaborated on in detail in Section 4.1.1 and Section 4.2.1. The part that could be scanned by Lidars was regarded as the visible part. The template point cloud should be dense to ensure the accuracy of point-to-point ICP fine registration.

**Figure 5:**
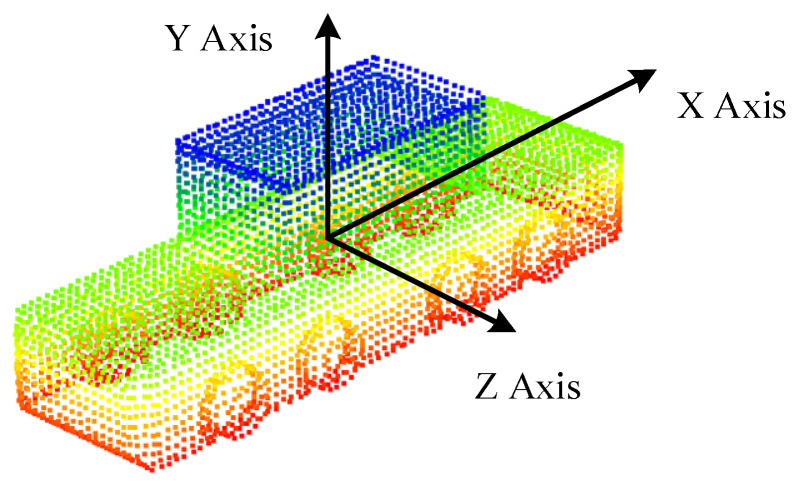
Target vehicle point cloud template aligned with the origin of the global coordinate system, which was elaborated on in detail in Section 4.1.1.

It is worth noting that the template point cloud needed to be aligned with the origin of the global coordinate system. The X-axis corresponded to the vehicle’s longitudinal direction. The Z-axis corresponded to the vehicle’s lateral direction, and the Y-axis corresponded to the vehicle’s vertical direction, as shown in Figure 5. This simplified the calculation of coordinate conversion between the template coordinate system and the global coordinate system. At this time, without translation and rotation transformation, the pose of the vehicle in the global coordinate system could be expressed as:

(6) $$\left[P_{xt},P_{yt},P_{zt},R_{xt},R_{yt},R_{zt}\right]=\left[0,0,0,1,0,0\right]$$

#### 3.3.2. ECPC Initial Pose Estimation

In the processing stage of ECPC-ICP, the initial pose estimation algorithm was needed to obtain a rough pose to provide a good initial value for the subsequent ICP fine registration. The experimental verification showed that the ICP algorithm had a higher tolerance for position errors and a lower tolerance for orientation errors. So the overall characteristics of the point cloud under road constraints were calculated to achieve a stable initial pose estimation. The ECPC initial pose estimation method in Figure 4 is described in detail as Algorithm 1.
**Algorithm 1** ECPC Initial Pose Estimation.**Input:** environment constraint vector $EC={\left[GN_{x},GN_{y},GN_{z}\right]}^{T}$, clustered point cloud $PC_{C}\in {\mathbb{R}}^{3\times N}$ **Output:** coarse rigid transform matrix $TM_{ECPC}\in {\mathbb{R}}^{4\times 4}$ **1:** $p_{max}\leftarrow \underset{p_{i}\in PC_{C}}{\max}\left\{p_{i}\right\}$ **2:** $p_{min}\leftarrow \underset{p_{i}\in PC_{C}}{\min}\left\{p_{i}\right\}$ **3:** $p_{center}\leftarrow \left(p_{max}+p_{min}\right)/2$ **4:** $\bar{PC_{C}}\leftarrow PC_{C}-p_{center}$ **5:** $cov\leftarrow \frac{1}{N}\bar{PC_{C}}\cdot {\bar{PC_{C}}}^{T}$ **6:** $u_{1}^{\prime }\leftarrow CalMaxEigenVectors\left(cov\right)$ **7:** $u_{2}\leftarrow Normalized\left(EC\times u_{1}^{\prime }\right)$ **8:** $u_{1}\leftarrow \left(u_{2}\times EC\right)$ **9:** $R\leftarrow \left[\begin{array}{ccc} GN_{x} & GN_{y} & GN_{z}\\ & u_{2}{}^{T} & \\ & u_{1}{}^{T} & \end{array}\right]$ **10: return** $\left[\begin{array}{cc} R & R\cdot p_{center}\\ \begin{array}{ccc} 0 & 0 & 0 \end{array} & 1 \end{array}\right]$

In Algorithm 1, since the shape of the target vehicle could be approximated as a rectangular parallelepiped, the calculated eigenvector and center under road constraints could meet the requirements of the initial pose. The result was less disturbed by target characteristics and noise points, which meant better robustness. The point cloud center was calculated in steps 1–3, and the eigenvector under environmental constraints was solved in steps 4–8. The rigid transformation matrix was integrated as output. Function CalMaxEigenVectors in step 6 was to calculate the eigenvector corresponding to the largest eigenvalue of the matrix. There were many mature solution methods, such as SVD decomposition [60]. The eigenvector was obtained by the eigendecomposition since cov was a real symmetric matrix in this paper. Function Normalized in step 7 was a vector normalization function, which converted the vector into a unit vector to ensure the rotation and translation invariance of the transformation. The R in step 9 represented the rotation transformation matrix of the ECPC initial pose, which was a component of the rigid transform matrix ${\mathrm{TM}}_{\mathrm{ECPC}}$.

Based on the rigid transformation matrix ${\mathrm{TM}}_{\mathrm{ECPC}}$ returned by Algorithm 1, this paper took the homogeneous form $\dot{PC_{T}}=\left[\begin{array}{cc} PC_{T} & 1 \end{array}\right]$ of the target template point cloud $PC_{T}$, and performed the rigid transformation to obtain an initial pose result matrix **IRM**:

(7) $$\mathrm{IRM}={\mathrm{TM}}_{\mathrm{ECPC}}\cdot \dot{PC_{T}}$$

#### 3.3.3. Precise Pose Calculation

At the stage of final pose calculation, the initial pose result IRM and the homogeneous form of the clustered point cloud $\dot{PC_{C}}=\left[\begin{array}{cc} PC_{C} & 1 \end{array}\right]$ were put into the classic point-to-point ICP algorithm to get the precise rigid transformation matrix ${\mathrm{TM}}_{\mathrm{ICP}}$:

(8) $${\mathrm{TM}}_{\mathrm{ICP}}=\left[\begin{array}{cc} R_{ICP\left[3\times 3\right]} & t_{ICP\left[3\times 1\right]}\\ 0_{\left[1\times 3\right]} & 1 \end{array}\right]$$

where $R_{ICP}$ and $t_{ICP}$ are respectively the rotation matrix and translation matrix solved by the ICP algorithm. Then the final 6D pose result matrix **FRM** was obtained:

(9) $$\mathrm{FRM}={\mathrm{TM}}_{\mathrm{ICP}}\cdot \mathrm{IRM}$$

So far, the rigid transformation matrix containing the 6D pose representation had been calculated:

(10) $$\left[\begin{array}{cc} R_{F} & t_{F}\\ \begin{array}{ccc} 0 & 0 & 0 \end{array} & 1 \end{array}\right]={\mathrm{TM}}_{\mathrm{ICP}}\cdot {\mathrm{TM}}_{\mathrm{ECPC}}$$

where $R_{F}\in {\mathbb{R}}^{3\times 3}$ and $t_{F}\in {\mathbb{R}}^{3}$, respectively, represent the final rotation matrix and translation matrix of the estimated vehicle pose. According to Equations (2), (3) and (6), the pose vector $\left[P_{x},P_{y},P_{z},R_{x},R_{y},R_{z}\right]$ could be derived. The specific calculation process was:

(11) $${\left[P_{x},P_{y},P_{z}\right]}^{T}=R_{F}\cdot {\left[P_{xt},P_{yt},P_{zt}\right]}^{T}+t_{F}=t_{F}$$

(12) $${\left[R_{x},R_{y},R_{z}\right]}^{T}=R_{F}\cdot {\left[R_{xt},R_{yt},R_{zt}\right]}^{T}=R_{F}\cdot {\left[1,0,0\right]}^{T}$$

## 4. Experiment

The validation of the proposed approach was addressed from two different perspectives. On the one hand, tests on a realistic simulated environment were performed to retrieve plentiful quantitative data with respect to the perfect ground truth. The performance of the proposed algorithm was verified by comparing it with existing methods under different test conditions. On the other hand, the method was also applied in a real environment to prove the validity of the approach in real use cases.

### 4.1. Simulated Test Environment

#### 4.1.1. Template Point Cloud Acquisition

**Figure 6:**
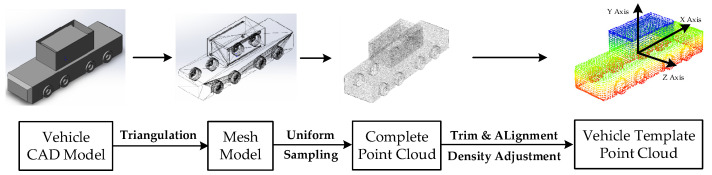
Template point cloud acquisition procedure in the simulated test environment.

The actual project’s centralized heavy-duty unmanned transport vehicle, depicted in Figure 1, was selected as the experimental target vehicle, whose size was 10.5 m×2.9 m×3.3 m. The template point cloud acquisition procedure is shown in Figure 6.

Based on the precise vehicle CAD model, a mesh model was obtained through triangulation. The complete point cloud was sampled from the mesh model and trimmed to exclude the invisible part. Then the vehicle template point cloud shown in Figure 5 was accomplished by density adjustment and alignment.

#### 4.1.2. Experimental Design

In the experimental verification stage, this paper compared the proposed algorithm to the model-free methods and model-based methods, including the PCA pose estimation method, the L-fitting [40] algorithm (with Closeness Criterion), the SHOT-ICP method (SAC-IA [35] with SHOT [25] descriptor and ICP) and FPFH-ICP algorithm (SAC-IA with FPFH [27] descriptor and ICP). An ablation study was also conducted to illustrate the contribution of the ECPC initial pose estimation component. The errors in the 6D pose estimation results provided by those under different measurement parameters were compared.

**Figure 7:**
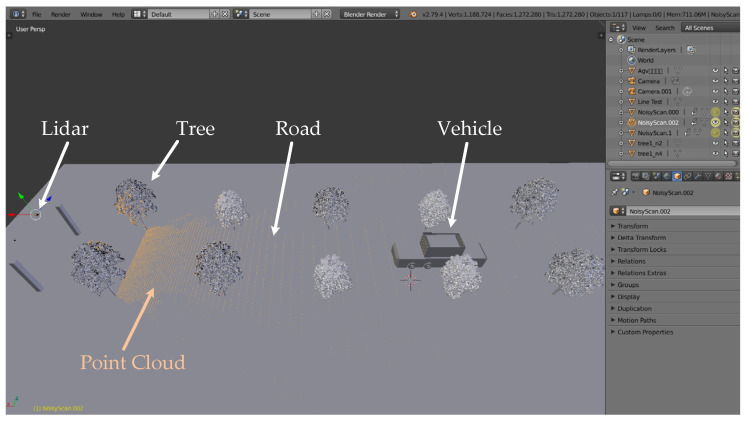
Sketch of the simulation scene and point cloud in BlenSor software (version 1.0.18).

The simulated environment model was built in SOLIDWORKS [61] according to our real test environment. Lidar point cloud data was obtained through simulation in BlenSor [62], which is shown in Figure 7. TOF Lidar was selected as the sensor to simulate most roadside measurement scenarios.

Since the proposed algorithm aimed at an accurate pose under sparse point clouds, this paper verified the performance of current algorithms for point clouds with different sparseness. Regarding the description of point cloud sparseness, the number of points was used in [63,64] to indicate the sparseness of the point cloud. Voxel size was adopted in [65,66] to distinguish point clouds with different sparseness. A multidimensional point cloud simplification function [67] was proposed to sparse the point cloud while retaining valid information. 

However, in the vehicle pose detection task using roadside Lidars, the point cloud volume and density of the vehicle varied with the vehicle pose, measurement distance, Lidar resolution, and measurement angle. Those point cloud sparseness descriptions could not accurately reflect the different sparseness effects due to distinct roadside measurement parameters.

**Figure 8:**
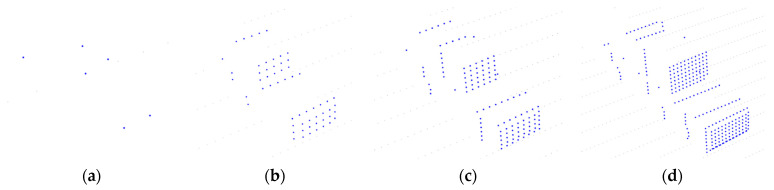
Vehicle point clouds under the same measurement angle with different sparseness. Blue points represent the target vehicle point cloud. (**a**) Point cloud with S=0.5 (6 points); (**b**) Point cloud with S=5 (59 points); (**c**) Point cloud with S=10 (126 points); (**d**) Point cloud with S=21 (274 points).

In this paper, to quantify the sparseness of the point cloud under different Lidar equipment and measurement conditions, the point cloud sparseness ***S*** was defined as the number of laser beams received on the unit area section from the center of the vehicle toward the position of the Lidar. The unit of ***S*** was $1/m^{2}$. Ignoring the effect of minimal angles, ***S*** could be calculated as:

(13) S=4(tan−112d)2resH·resV

where d is the perception distance, that is, the distance between the center of the vehicle and the center of the Lidar. $res_{H}$ and $res_{V}$ are the vertical and horizontal laser resolution of the Lidar, respectively. For example, the ***S*** of Velodyne VLP-16 (10 Hz) at 30 m was 9.1, and the ***S*** of Velodyne HDL-64E (10 Hz) at 30 m was 57. Under the same conditions, the data volume of HDL-64E was about six times that of VLP-16. The vehicle point clouds under the same measurement angle with different sparseness are displayed in Figure 8, which proved that S could well describe the sparseness caused by various measurement conditions. The point clouds were simulated in BlenSor.

In this paper, considering general roadside measurement scenarios, the range of ***S*** for the simulation was [0, 22]. Under the same ***S***, the statistical results of poses in all situations were used as experimental values. At the same time, a Gaussian measurement error $E\sim N\left(0,0.005\;m^{2}\right)$ was applied to the point cloud to simulate the measurement error of the Lidar itself, which better tested the algorithms under all working conditions. The ground truth poses of the vehicle were obtained directly from the simulation parameters.

#### 4.1.3. Results and Discussion

**Figure 9:**
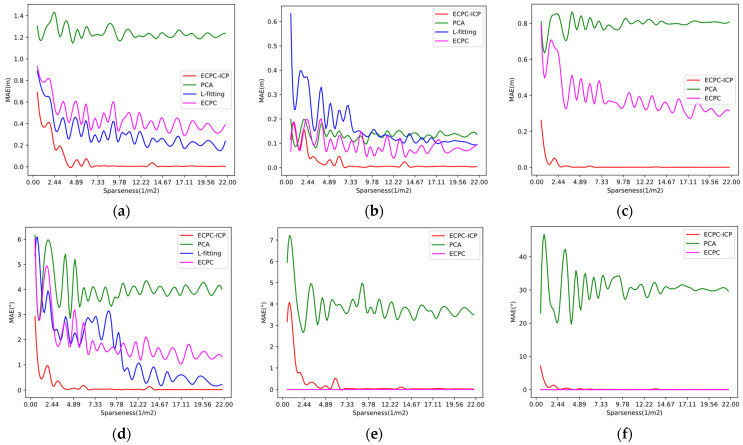
MAE of different algorithms under point clouds with different sparseness. (**a**) MAE of different algorithms on local *X*-axis; (**b**) MAE of different algorithms on local *Y*-axis; (**c**) MAE of different algorithms on local *Z*-axis; (**d**) MAE of different algorithms on local yaw angle; (**e**) MAE of different algorithms on local pitch angle; (**f**) MAE of different algorithms on local roll angle.

The vehicle coordinate system MAE (Mean Absolute Error) of the proposed algorithm was compared with PCA and L-fitting algorithms under point clouds of different sparseness. Their MAE of different dimensions ($\left[P_{x},P_{y},P_{z},R_{x},R_{y},R_{z}\right]$) were calculated in the ground truth pose coordinate system. The ECPC initial pose error was also calculated for the ablation study. The results are shown in Figure 9. The MAE in all cases was calculated, as shown in Table 1. Since the L-fitting algorithm operated on a two-dimensional plane and could only solve the three-degree-of-freedom vehicle pose, its results only appeared in (a), (b), and (d) of Figure 9.

Figure 9 and Table 1 show that ECPC initial pose result was relatively accurate and stable, providing a good foundation for the subsequent ICP registration. By fusing road constraints, ECPC achieved a robust initial pose estimation under sparse point cloud conditions. Because the vehicle drove perfectly on the road surface in the simulation, the pitch error and roll error of the ECPC pose was close to 0.

As shown in Figure 9 and Table 1, the 6D pose estimation accuracy of ECPC-ICP was better than the competing algorithms. Even in the case of extremely sparse point clouds, ECPC-ICP could achieve relatively accurate measurements. For the error distribution, when the point cloud sparseness was normal, PCA could obtain a relatively stable result faster. L-fitting could obtain a stable and accurate two-dimensional yaw angle, and ECPC-ICP could solve a stable and precise 6D pose.

As the sparseness ***S*** of the input point cloud decreased, the MAE of the three algorithms had increased in different ranges. The internal reasons were various. For PCA, the sparse point cloud reduced the percentage of effective points in the point cloud, and the description of the vehicle shape by the point cloud decreased. Random error points caused significant interference to the statistical results, increasing the overall pose MAE. L-fitting relied on the vehicle boundary points. The sparse points reduced the proportion of boundary points, thereby reducing the description of the vehicle boundary shape. Some vehicle central point clouds and random noise could also produce morphological interference, which increased the probability of misjudgment of vehicle attitude and caused an increase in MAE.

For ECPC-ICP, due to global point-to-point registration, the sparse vehicle point cloud had to have excellent global distribution. As the point cloud gradually became sparse, the local description was destroyed, while the proportion of local points increased and the global distribution decreased. As a result, ECPC-ICP was easier to converge to the local optimum in the registration stage, leading to increased MAE. Therefore, although the accuracy of the proposed algorithm went down as ***S*** decreased, it still achieved better results than the competing methods. 

**Figure 10:**
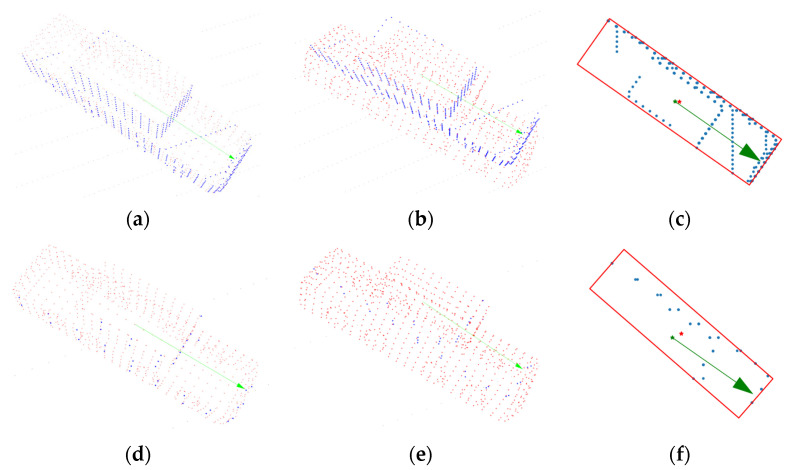
Typical pose estimation results of different methods, where blue points represent the measurement data and red points represent the estimated pose results. Green arrows represent the ground truth (arrow starting point represents the ground truth location, and the arrow direction represents ground truth orientation). (**a**) ECPC-ICP with S=20; (**b**) PCA with S=20; (**c**) L-fitting with S=20; (**d**) ECPC-ICP with S=2; (**e**) PCA with S=2; (**f**) L-fitting with S=2.

For a more intuitive explanation, the typical situation of the experiment is shown in Figure 10. The closer the blue points were to the vehicle surface represented by the red points, the higher was the accuracy of the pose estimation result. It indicated that ECPC-ICP achieved accurate pose results with S=20 and S=2. L-fitting estimated an accurate pose with S=20, but had a small orientation error with S=2. The results of PCA were relatively inaccurate.

**Figure 11:**
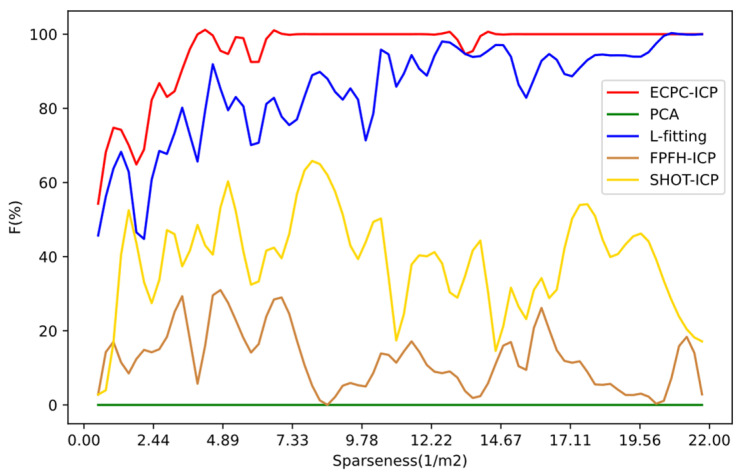
Pose estimation success ratio ***F*** of ECPC-ICP in different cases compared with other methods.

Furthermore, to verify the robustness of the proposed algorithm, this paper compared the pose estimation success ratio of ECPC-ICP, PCA, L-fitting, SHOT-ICP, and FPFH-ICP in all test data. The pose estimation success ratio ***F*** was expressed explicitly as:

(14) $$F=\frac{N_{AC}}{N_{all}}$$

where $N_{AC}$ is the number of test samples whose error is less than the threshold, and $N_{all}$ is the total number of test samples. The results under different sparseness are shown in Figure 11, and the statistical results are shown in Table 2.

As shown in Figure 11 and Table 2, the proposed ECPC-ICP showed better robustness under different sparse point clouds. L-fitting could also solve reasonable three-dimensional vehicle pose results in most cases. Feature-based FPFH and SHOT descriptors could obtain stable poses in dense point clouds, but the estimation of poses in the case of sparse point clouds was unstable. The SHOT descriptor was slightly better than FPFH because of its better description of local features. Although PCA could hardly solve the accurate vehicle pose, meaningful statistical characteristics of the target point cloud were obtained, which could play a significant role in rough calculation and estimation.

As the sparseness of the point cloud decreased, the robustness of each algorithm had a downward trend. In the sparsest test data with an ***S*** of 0.5, the SHOT and FPFH calculations failed. The success ratio ***F*** of L-fitting was about 46%, and the success ratio ***F*** of ECPC-ICP was about 55%, which still guaranteed availability. In all the test data, the total success ratio ***F*** of the proposed algorithm was 95.5026%, which showed better robustness for sparse point clouds than the competing methods.

**Figure 12:**
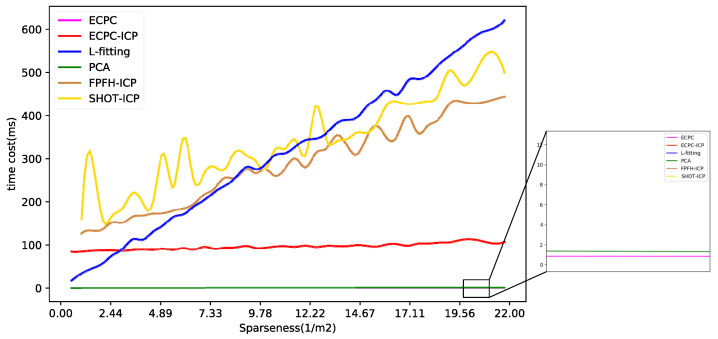
The calculation time distribution of all methods.

The calculation time distribution of all methods is shown in Figure 12, and the average time cost is shown in Table 3. As the amount of input data increased, the time consumption of all algorithms increased to varying degrees. The calculation time of L-fitting, FPFH-ICP, and SHOT-ICP had larger increases due to their high computational complexity. The calculation time of ECPC, ECPC-ICP, and PCA was relatively stable, verifying higher robustness.

In general, the calculation time of ECPC and PCA was extremely short, while effective point cloud information could be quickly obtained. The calculation time of FPFH-ICP and SHOT-ICP was relatively long due to their complexity in local feature computation. The calculation time of L-fitting varied greatly with point cloud sparseness, showing weak stability. The mean calculation time of ECPC-ICP was 96.13 ms, basically meeting the needs of real-time pose estimation.

### 4.2. Real Test Environment

#### 4.2.1. Template Point Cloud Acquisition

**Figure 13:**
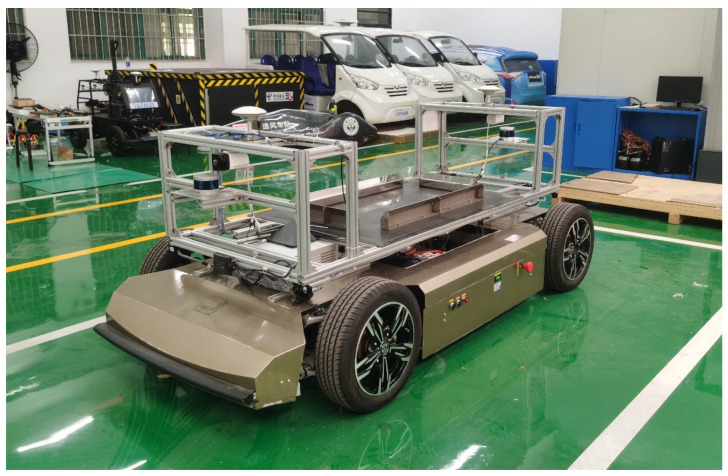
The functional experimental vehicle used in experimental verification.

Limited to unfinished heavy-duty transport vehicle manufacturing, a functional vehicle with similar geometric shapes was selected in the real environment test, which is shown in Figure 13. The vehicle size was 3 m×1.4 m×1.3 m.

**Figure 14:**
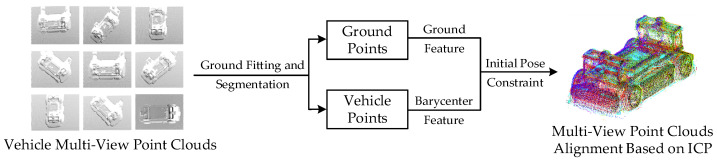
The splicing procedure for template point cloud acquisition.

The template Point Cloud was obtained by splicing from the scanned multi-view vehicle point clouds. The number of multi-view vehicle point clouds should be appropriate to balance the accuracy of splicing and the simplicity of the process. Ground features were also used to achieve the multi-view pose initialization. Our splicing procedure is shown in Figure 14.

**Figure 15:**
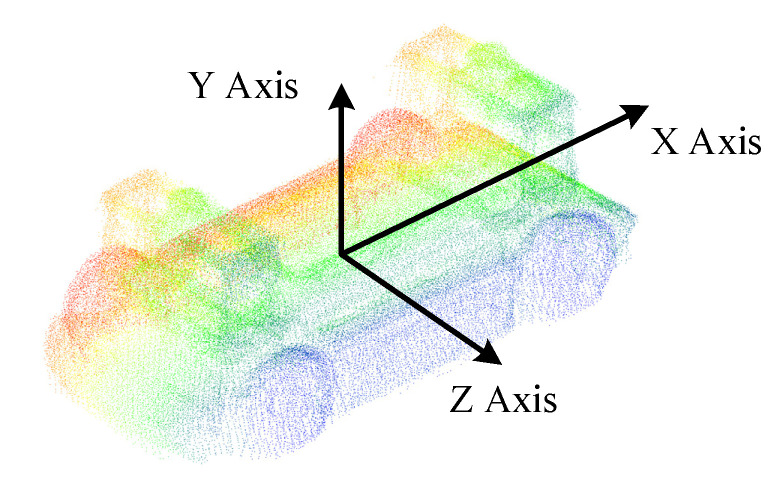
The functional vehicle point cloud template aligned with the origin of the global coordinate system.

In this paper, the number of multi-view vehicle point clouds was set to 9, ensuring a 50% overlap between the adjacent point clouds. The ground normal features and vehicle barycenter features were extracted for pose initialization. The template point cloud after ICP alignment is shown in Figure 14, where different colors represent point clouds from different perspectives. After density adjustment and aligning with the origin of the global coordinate system, the completed template point cloud of the experimental vehicle is shown in Figure 15.

#### 4.2.2. Experimental Design

**Figure 16:**
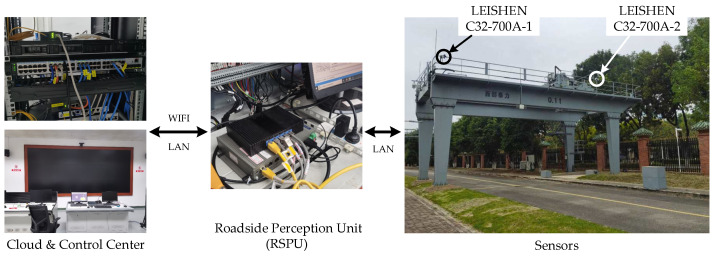
The roadside Lidar measurement system.

Based on the proposed algorithm’s accuracy and robustness verified by the simulation experiment, the method was also applied in the real environment to prove the validity in real use cases. The roadside Lidar measurement system, depicted in Figure 1, was built based on two LEISHEN C32-700A [68] Lidars, as shown in Figure 16.

Under different measurement distances and vehicle attitudes, about 700 frames of roadside point cloud data were collected to verify the feasibility of ECPC-ICP. Furthermore, to verify the accuracy of the algorithm in the real test environment, the GNSS/RTK pose of the vehicle-mounted positioning system was used as a reference to calculate the 6-dimensional error of the ECPC-ICP pose.

Specifically, ten measurement positions were set at different measurement distances and vehicle poses. For each measurement position, the average value of GNSS/RTK data within 30 s was calculated as the GNSS/RTK reference pose. The six-dimensional pose error was calculated in the GNSS/RTK pose coordinate system. Referring to the concept of the ICP loss function, the average closest point error was also calculated to compare the accuracy of GNSS/RTK pose and ECPC-ICP pose. The expression was as follows:

(15) $$L_{GNSS}=\frac{1}{N}\overset{N}{\underset{i=1}{\sum }}\left\|p_{source}^{i}-p_{GNSS}^{i}\right\|$$

(16) $$L_{F}=\frac{1}{N}\overset{N}{\underset{i=1}{\sum }}\left\|p_{source}^{i}-p_{F}^{i}\right\|$$

where $L_{GNSS}$ and $L_{F}$ represent the average closest point error of GNSS/RTK pose and ECPC-ICP pose, respectively. $p_{source}^{i}$ represents the points in the clustered point cloud $PC_{C}$. N represents the points’ number of $PC_{C}$. $p_{GNSS}^{i}$ and $p_{F}^{i}$ represent the nearest point of $p_{source}^{i}$ in the template point cloud under GNSS/RTK pose and ECPC-ICP pose, respectively.

It is worth mentioning that the point cloud sparseness S had different meanings for vehicles of different scales. For larger-scale vehicles, the vehicle point cloud had more points under the same sparseness, which made it easier to estimate the vehicle pose. In this subsection, due to the smaller size of the functional vehicle, in order to be consistent with Section 4.1, equivalent sparseness $S^{\prime }$ was used instead of absolute sparseness S:

(17) $$S^{\prime }=\frac{\mathrm{heavy}-\mathrm{duty}\;\mathrm{vehicle}\;\mathrm{size}}{\mathrm{functional}\;\mathrm{vehicle}\;\mathrm{size}}S=0.15\cdot S$$

#### 4.2.3. Results and Discussion

**Figure 17:**
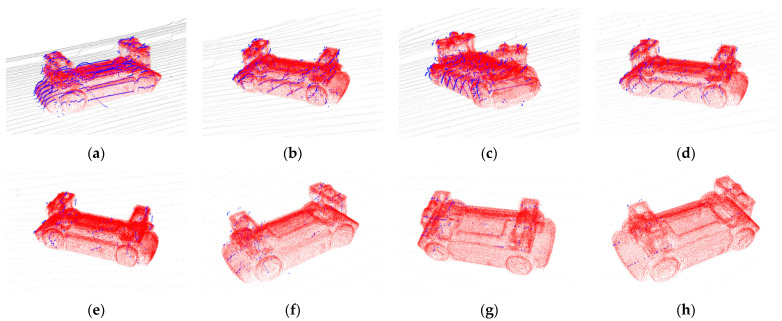
Typical pose estimation results in the real test environment. Blue points represent the clustered point cloud. Black points represent the background points, and red ones represent estimated pose results. (**a**) $S^{\prime }=22.65$; (**b**) $S^{\prime }=15.62$; (**c**) $S^{\prime }=12.58$; (**d**) $S^{\prime }=8.5$; (**e**) $S^{\prime }=4.4$; (**f**) $S^{\prime }=4.67$; (**g**) $S^{\prime }=4.2$; (**h**) $S^{\prime }=2.8$.

The typical pose estimation results of the proposed algorithm in the real test environment are shown in Figure 17, which illustrated the accuracy and reliability under different vehicle pose and point cloud sparseness.

The closer the blue points were to the vehicle surface represented by the red points, the higher was the accuracy of the pose estimation result. In a sparse point cloud scene, the proposed ECPC-ICP still showed high stability. Based on reasonable point cloud distribution, a stable 6D pose could be achieved under the condition of more noise points and fewer input points, verifying the effectiveness of the proposed algorithm. 

Furthermore, the comparison results of ECPC-ICP pose and GNSS/RTK pose are shown in Table 4, where the 6D error represents the x-axis error, y-axis error, z-axis error, yaw error, pitch error, and roll error.

The experimental result showed that the average positioning error of ECPC-ICP pose results was 0.08 m, which was 2.6% of the vehicle scale, and the average orientation error was 1.64°. The average closest point error of GNSS/RTK pose was 0.0230 m. The average closest point error of ECPC-ICP pose was 0.0188, which was about 0.8 times that of the GNSS/RTK pose. Although the average closest point error *L* could not directly describe the accuracy of the pose, the smaller *L* in the same scene reflected higher accuracy.

The result proved that the ECPC-ICP pose had the same or even higher accuracy than the GNSS/RTK pose. Unlike GNSS/RTK poses that might be interfered by obstruction or weather, the proposed ECPC-ICP pose estimation method could work stably in almost all situations, including GNSS denied indoor or tunnel scenes.

**Figure 18:**
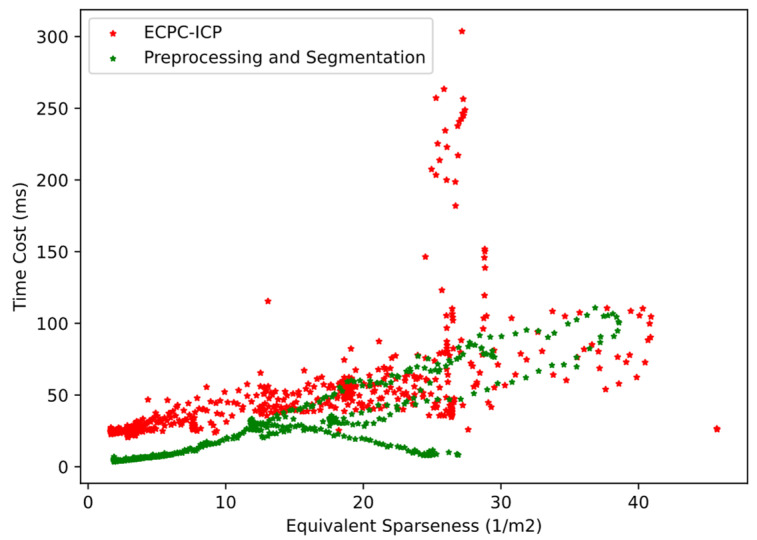
The calculation time cost distribution of ECPC-ICP and the preprocessing and segmentation module in all real environment tests.

**Figure 19:**
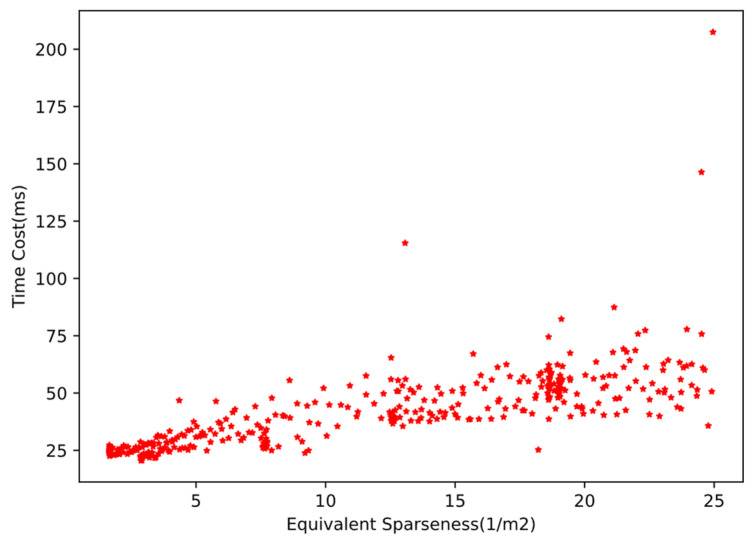
The calculation time cost distribution of ECPC-ICP of $S^{\prime }$ from 0 to 25.

The calculation time cost distribution of ECPC-ICP in about 700 frames point cloud data is shown in Figure 18. The calculation time cost distribution of the preprocessing and segmentation module is also shown in Figure 18. The time cost distribution of ECPC-ICP with $S^{\prime }$ from 0 to 25 is shown in Figure 19, which represents the time cost distribution in the general roadside measurement scene. The statistical results of time cost are shown in Table 5.

The calculation time cost of the preprocessing and segmentation module varied with the number of foreground points. The mean time cost was 27.82 ms, verifying its effectiveness.

The calculation time cost of ECPC-ICP varied with the vehicle attitude and point cloud sparseness. Experimental results showed that the ECPC-ICP pose estimation was relatively fast in most scenarios. The average FPS was 18.8 Hz in the real test environment, and was 24.8 Hz with $S^{\prime }<25$ verifying the effectiveness of the proposed method in practical applications.

## 5. Conclusions

Target vehicle pose estimation based on roadside Lidar is a crucial issue in the cooperative perception of intelligent vehicles. This paper combined the idea of model-free and model-based pose estimation methods. Aiming at the sparse point cloud characteristics, an ECPC-ICP algorithm for accurate 6D pose estimation was proposed. After the point cloud was preprocessed, filtered, and clustered, the initial pose was obtained by fusing the road feature constraint and the eigenvector information in the ECPC algorithm. The accurate 6D pose estimation was obtained through a point-to-point ICP algorithm with a vehicle point cloud template. 

In addition, the sparseness ***S*** of the observation point cloud was defined according to the roadside Lidar measurement characteristics. Through simulated experiment testing under different sparseness point cloud conditions, comparing the calculation results of ECPC-ICP, PCA, L-fitting, SHOT-ICP, and FPFH-ICP, the proposed algorithm had the same accuracy as the current algorithms under good point cloud sparseness conditions. Under relatively sparse point cloud conditions, the proposed algorithm had greater accuracy and robustness than competing methods. Under extremely sparse point cloud conditions, the ECPC-ICP could still maintain a certain degree of usability. 

The results in the real test environment showed that the average positioning error of ECPC-ICP pose results was 0.08 m, which was 2.6% of the vehicle scale, and the average orientation error was 1.64°. The mean time cost was 53.19 ms. The experiments proved the accuracy and effectiveness of ECPC-ICP in real test environments. This paper provided a more accurate and robust pose solution for cooperative perception, which could work stably in almost all situations, including GNSS-denied indoor or tunnel scenes. This paper also provided some exploration for roadside equipment layout in cooperative perception cases.

As future work, we will expand the recognition model to achieve multi-target pose recognition and tracking and explore the information interchange between vehicles and roadside facilities to achieve a higher level of cooperative perception.

## Figures and Tables

**Table 1:** MAE of different methods in all cases.

Method	Error MAE (m)			Error MAE (deg)		
	Local *X*-Axis	Local *Y*-Axis	Local *Z*-Axis	Yaw	Pitch	Roll
PCA	1.23932	0.13477	0.79728	4.07111	3.89515	30.49411
L-fitting	0.31533	0.17401	/	1.58945	/	/
ECPC (Ours)	0.46180	0.09200	0.39429	1.96267	0.00042	0.00044
ECPC-ICP (Ours)	**0.06334**	**0.02157**	**0.01066**	**0.16794**	**0.27018**	**0.34759**

**Table 2:** Pose estimation success ratio ***F*** of different methods in all cases.

Method	Success Ratio *F*
PCA	2.7777%
L-fitting	84.7222%
FPFH-ICP	14.7487%
SHOT-ICP	40.4762%
ECPC-ICP (Ours)	**95.5026%**

**Table 3:** The mean calculation time of all methods.

Method	Mean Calculation Time (ms)
PCA	0.8135
L-fitting	308.24
FPFH-ICP	283.11
SHOT-ICP	335.77
ECPC (Ours)	0.4633
ECPC-ICP (Ours)	96.13

**Table 4:** The comparison results of ECPC-ICP pose and GNSS/RTK reference pose.

Index	ECPC-ICP Pose	GNSS/RTK Pose	6D Error (m&°)	$S^{\prime }$ (1/m^2^)	$L_{GNSS}$ (m)	$L_{F}$ (m)
1	(4.718, −6.426, 5.609, 0.922, −0.003, 0.386)	(4.659, −6.456, 5.739, 0.917, −0.008, 0.397)	(0.002, 0.026, −0.143, −0.65, 0.27, 1.78)	17.1	0.0198	0.0112
2	(9.310, −6.418, 5.141, 0.999, −0.009, −0.018)	(9.322, −6.457, 5.161, 0.999, −0.002, −0.032)	(−0.011, 0.038, −0.021, 0.78, −0.36, 1.84)	10.4	0.0176	0.0137
3	(10.967, −6.401, 5.727, 0.839, 0.013, 0.543)	(10.990, −6.413, 5.650, 0.855, −0.013, 0.518)	(0.020, 0.014, 0.077, 1.67, 1.55, −1.41)	8.4	0.0171	0.0120
4	(14.572, −6.421, 4.086, −0.041, −0.015, −0.999)	(14.548, −6.377, 4.086, −0.046, 0.0003, −0.998)	(−0.0005, −0.043, 0.024, 0.34, −0.88, 0.78)	6.05	0.0226	0.0170
5	(9.248, −6.407, 5.567, 0.828, 0.001, 0.559)	(9.210, −6.387, 5.564, 0.831, −0.013, 0.556)	(0.033, −0.019, −0.018, 0.26, 0.87, −0.069)	10.35	0.0134	0.0097
6	(17.988, −6.357, 5.991, 0.996, 0.077, 0.005)	(18.117, −6.406, 5.80, 0.997, −0.0003, −0.077)	(−0.143, 0.056, 0.178, 4.59, 4.67, −10.81)	4.09	0.0698	0.0703
7	(8.371, −6.411, 4.195, 0.998, 0.004, −0.058)	(8.336, −6.404, 4.240, 0.997, −0.002, −0.077)	(0.037, −0.007, −0.042, 1.05, 0.39, 0.50)	12.6	0.0119	0.0089
8	(10.418, −6.401, 5.180, 0.588, −0.0008, 0.808)	(10.401, −6.375, 5.213, 0.587, −0.019, 0.808)	(−0.016, −0.027, −0.032, −0.07, 1.06, −1.62)	9.3	0.0154	0.0097
9	(8.484, −6.407, 4.404, 0.098, −0.0037, −0.995)	(8.465, −6.409, 4.481, 0.084, −0.0019, −0.996)	(0.078, 0.0016, 0.012, 0.81, −0.08, 4.62)	12.3	0.0163	0.0113
10	(18.851, −6.375, 4.479, 0.999, 0.036, −0.0025)	(18.880, −6.398, 4.503, 0.999, −0.003, −0.028)	(−0.028, 0.023, −0.026, 1.38, 2.26, 0.75)	3.9	0.0119	0.0104

**Table 5:** The statistical results of ECPC-ICP time cost and the preprocessing and segmentation module time cost.

Method	Mean Time Cost (ms)	Mean Time Cost $\left(S^{\prime }<25\right)$ (ms)
ECPC-ICP	53.1928	40.3334
Preprocessing and Segmentation	27.8227	/

## Data Availability

Not applicable.
